# PTEN Loss Increases PD-L1 Protein Expression and Affects the Correlation between PD-L1 Expression and Clinical Parameters in Colorectal Cancer

**DOI:** 10.1371/journal.pone.0065821

**Published:** 2013-06-13

**Authors:** Minmin Song, Defeng Chen, Biyan Lu, Chenliang Wang, Junxiao Zhang, Lanlan Huang, Xiaoyan Wang, Christine L. Timmons, Jun Hu, Bindong Liu, Xiaojian Wu, Lei Wang, Jianping Wang, Huanliang Liu

**Affiliations:** 1 Institute of Gastroenterology and the Sixth Affiliated Hospital, Sun Yat-sen University, Guangzhou, Guangdong, China; 2 Institute of Human Virology, Sun Yat-sen University, Guangzhou, Guangdong, China; 3 Key Laboratory of Tropical Disease Control (Sun Yat-sen University), Ministry of Education, Guangzhou, Guangdong, China; 4 Dongguan Health School, Dongguan, Guangdong, China; 5 Center for AIDS Health Disparities Research, Department of Microbiology and Immunology, Meharry Medical College, Nashville, Tennessee, United States of America; Rush University Medical Center, United States of America

## Abstract

**Background:**

Programmed death ligand-1 (PD-L1) has been identified as a factor associated with poor prognosis in a range of cancers, and was reported to be mainly induced by PTEN loss in gliomas. However, the clinical effect of PD-L1 and its regulation by PTEN has not yet been determined in colorectal cancer (CRC). In the present study, we verified the regulation of PTEN on PD-L1 and further determined the effect of PTEN on the correlation between PD-L1 expression and clinical parameters in CRC.

**Methods/Results:**

RNA interference approach was used to down-regulate PTEN expression in SW480, SW620 and HCT116 cells. It was showed that PD-L1 protein, but not mRNA, was significantly increased in cells transfected with siRNA PTEN compared with the negative control. Moreover, the capacity of PTEN to regulate PD-L1 expression was not obviously affected by IFN-γ, the main inducer of PD-L1. Tissue microarray immunohistochemistry was used to detect PD-L1 and PTEN in 404 CRC patient samples. Overexpression of PD-L1 was significantly correlated with distant metastasis (P<0.001), TNM stage (P<0.01), metastatic progression (P<0.01) and PTEN expression (P<0.001). Univariate analysis revealed that patients with high PD-L1 expression had a poor overall survival (P<0.001). However, multivariate analysis did not support PD-L1 as an independent prognostic factor (P = 0.548). Univariate (P<0.001) and multivariate survival (P<0.001) analysis of 310 located CRC patients revealed that high level of PD-L1 expression was associated with increased risks of metastatic progression. Furthermore, the clinical effect of PD-L1 on CRC was not statistically significant in a subset of 39 patients with no PTEN expression (distant metastasis: P = 0.102; TNM stage: P = 0.634, overall survival: P = 0.482).

**Conclusions:**

PD-L1 can be used to identify CRC patients with high risk of metastasis and poor prognosis. This clinical manifestation may be partly associated with PTEN expression.

## Introduction

Colorectal cancer (CRC) is the second leading cause of cancer deaths in western countries. Globally, it is the third most commonly diagnosed cancer in males and the second most commonly diagnosed cancer in females [1]. What’s worse is that the mortality of CRC continues to increase in many countries with metastatic colorectal cancer (mCRC) being the primary cause of death in most patients. Early stage CRC patients had a five-year survival rate of up to 90%, but the rate could drop to 60% amount patients with lymph node involvement, and even down to 10% when metastases are present [2]. However, it’s difficult to detect the mCRC at early stage only based on symptoms, which leads to complicated treatment decision making, often including aggressive therapy or treatment holidays. A biomarker that can identify patients with high risk of metastasis and poor prognosis could have broad clinical applications. Studies that search for such biomarkers are abundant. PD-L1 is one of the major subjects of biomarker research.

PD-L1 (also known as CD274, B7-H1), one of the ligands for programmed cell death 1 (PD-1), is an immune-inhibitory receptor belonging to CD28/cytotoxic T lymphocyte antigen 4 (CTLA-4) family. It can deliver an inhibitory signal to PD-1/B7-1 expressing T cells, resulting in immune impairment [3], [4]. PD-L1 protein is often expressed on activated T cells, B cells, NK cells, DCs, macrophages, and bone marrow-derived mast cells [5]. PD-L1 expression is also found on a wide range of human tumours. In addition, studies relating PD-L1 expression to disease outcome have been done in most tumours, and the results show that PD-L1 expression strongly correlates with unfavourable prognosis in kidney [6], ovarian [7], bladder [8], breast [9], liver [10], gastric [11], and pancreatic cancer [12], but not in non-small cell lung cancer (NSCLC) [13]. Most importantly, these studies reveal that higher expression of PD-L1 may facilitate advancement of tumour stage and increase the invasion potential. However, in CRC, the clinical effect of PD-L1 and its regulation mechanism has not yet been determined.

PD-L1 expression can be induced by many inflammatory mediators and cytokines, of which Interferon-γ (IFN-γ) is the most potent. It has been shown that both type I and type II IFNs upregulate PD-L1 expression in most cancer cell lines. Few studies focus on the regulatory role of PD-L1 in tumors. Moreover, these studies produced divergent results. A recent study indicated that the loss of tumor suppressor PTEN (phosphatase and tensin homolog deleted on chromosome ten) may be an important mechanism that increases PD-L1 expression in glioma cell lines and patient tumor specimens [14]. PTEN is an important tumour-suppressor gene which primarily negatively regulates the cell-survival signaling PI3K-protein kinase B (Akt) pathway [15], [16]. In addition, accumulating studies showed that PTEN may be an important gene that associated with tumor metastasis [15], [17], [18]. Both in *vitro* and in *vivo* experiments of CRC showed that PTEN controls the tumorigenic and metastatic potential. It is reported that injection of PTEN-deficient cells in mice resulted in much more pulmonary and “multi-location” metastases than injection of control cells [19]. Furthermore, some clinical data indicates that loss of detection of PTEN by immunostaining was associated with advanced disease, liver metastasis and poor patient survival [20]–[22]. However, some recent studies do not support the role of PTEN in regulation of PD-L1 expression. It was said that “PD-L1 expression was modulated through different pathways among different tumor cell types” [23].

It is well known that PD-L1 is a potent negative regulator of antitumor immunity, but it is unknown whether this immune-escaping effect is sufficient to make PD-L1 a distinct factor associated with poor prognosis for all types of human cancers. Conversely, PTEN has been identified as a crucial factor in various central processes of cancer development, and as an important gene for cancer diagnosis and prognosis. Following the recent finding that PD-L1 protein expression correlated with PTEN loss [14], we came to the hypothesis that tumour progression and PD-L1 expression are independently related to PTEN loss, and that the clinical effect of PD-L1 may, at least in part, be attributed to the correlation between PTEN loss and PD-L1 expression.

In the present study, we first examined the effect of PTEN loss on PD-L1 expression in CRC cell lines, and determined whether the observed effect maintained in the presence of IFN-γ. Second, we assessed the association of PD-L1 expression with disease outcome and PTEN expression in 404 CRC patients with a median follow-up of 5 years. Lastly, we investigated the clinical significance of PD-L1 in a subset of 39 patients with PTEN complete loss to explore whether the effect of PD-L1 in CRC is dependent on PTEN expression.

## Materials and Methods

### Ethics Statement

This study was approved by the institutional review boards of Sun Yat-Sen University (Guangzhou, China), and written informed consent was obtained from each patient.

### Sample Collection and Cell Culture

In this study, 404 formalin-fixed, paraffin-embedded samples of CRC were obtained from the tumour bank of the Department of Pathology of the First Affiliated Hospital, Sun Yat-Sen University (Guangzhou, China). These 404 patients who had been diagnosed with CRC and underwent initial surgical resection for CRC between January 2000 and November 2006 were follow-up by telephone or letters from surgery up until April 2010 to collect general information, pathology reports, and information regarding the patients’ condition after surgery.

Three CRC cell lines, SW480, SW620 and HCT116 were purchased from Culture Collection of Chinese Academy of Science (Shanghai, China), and cultured in RPMI 1640 supplemented with 10% FBS and 1% penicillin-streptomycin at 37°C in 5% CO_2_.

### RNA Interference (RNAi)

To down-regulate PTEN expression, the specific siRNA duplexes targeting human PTEN (sense: 5′-GAGCGUGCAGAUAAUGACAdTdA-3′; antisense: 3′-dAdTCUCGCACGUCUAUUACUGU-5′) were synthesized and purchased from RiboBio Co. Ltd (Guangzhou, China), and siRNA duplexes with non-specific sequences were used as negative control (NC). SiRNA transfection was carried out with lipofectamine 2000 (Invitrogen, CA, USA) with a concentration of 100 nM. Transfection efficiency was tested by qRT-PCR and western blot. These experiments were performed in the presence and absence of recombinant human IFN-γ (500 IU/mL, Peprotech, NJ, USA) and repeated in triplicates.

### Quantitative Reverse Transcription PCR (qRT-PCR)

Forty-eight hours post transfection, total cellular RNA was extracted with TRIzol reagent (Invitrogen, CA, USA) according to the manufacturer’s protocol. cDNA was derived from 500 ng of total RNA using cDNA reverse transcription kit (Toyobo, Osaka, Japan). Afterwards, real time PCR was performed using the quantitative SYBR Green PCR kit (TaKaRa Bio, Dalian, China) on ABI 7500 RT-PCR System (Applied Biosystems, CA, USA) according to the manufacturer’s protocol. The primers used are listed in Table 1, PTEN and PD-L1 relative gene expression level was analyzed using the comparative C_T_ method (also referred to as the 2^−ΔΔCT^ method). Details of the comparative C_T_ method have been previously described [24], [25]. Briefly, three replicate PCR amplifications were performed for each sample. β-actin was used as an internal control. The average C_T_ was calculated for both the target genes and β-actin. The ΔC_T_ was determined as (the mean of the triplicate C_T_ values for the target gene) minus (the mean of the triplicate C_T_ values for β-actin). The ΔΔC_T_ represented the difference between the paired cell lines, where PTEN ΔΔC_T_ = ΔC_T_ of cells transfected with siRNA minus ΔC_T_ of negative control and PD-L1 ΔΔC_T_ = ΔC_T_ of cells treated with IFN-γ minus ΔC_T_ of the untreated control cells. The relative expression level was expressed as 2^−ΔΔCT^.

**Table 1:** Primers used in quantitative reverse transcription PCR (qRT-PCR).

Name	Primer sequence forward	Primer sequence reverse
**PTEN**	5′-AGGGACGAACTGGTGTAATGA-3	5′-CTGGTCCTTACTTCCCCATAGAA-3′
**PD-L1**	5′-TGGCATTTGCTGAACGCATTT-3′	5′-TGCAGCCAGGTCTAATTGTTTT-3′
**β-actin**	5′-CAATGAGCTGCGTGTGGCT-3′	5′-TAGCACAGCCTGGATAGCAA-3′

### Western Blot

Seventy-two hours post transfection, cells were washed three times with phosphate-buffered saline (PBS) and lysed with RIPA buffer (Dingguo, Beijing, China) supplemented with protease and phosphatase inhibitors (Merck Bioscience, Darmstadt, Germany). Afterwards, PTEN protein levels were determined using two-color fluorescent western blotting on the Odyssey infrared imaging system (LI-COR, Nebraska, USA) as previously described [26]. In brief, equal amounts of protein were separated by 10% sodium dodecyl sulphate-polyacrylamide gel electrophoresis (SDS-PAGE) and transferred to a polyvinylidene fluoride (PVDF) membrane (Pall, New York, USA). Membranes were then blocked with 5% BSA for 1 h and incubated with primary antibodies over night at 4°C [Primary antibody: Rabbit anti-PTEN (diluted 1∶10000, Epitomics, CA, USA); Rabbit anti-Akt (diluted 1∶1000, Cell Signaling Technology, MA, USA); Rabbit anti-phospho-Akt^Ser473^(diluted 1∶1000, Cell Signaling Technology, MA, USA); Mouse β-actin antibody (diluted 1∶10000, Proteintech, Chicago, USA)]. The next day, after incubating with species-appropriate fluorescently conjugated secondary antibodies for 1 h at room temperature, the membranes were analyzed using the Odyssey infrared imaging system.

### Flow Cytometry

PD-L1 expression on the cell surface was determined using Flow cytometers. Cells were harvested and incubated with anti-human PD-L1 PE-Cy7 (eBioscience, CA, USA) or isotype-matched control antibody (eBioscience, CA, USA) for 30 minutes in the dark on ice (0.5 µg antibodies for 10^5^ cells in a final volume of 100 µL). After samples were washed three times, stained cells were re-suspended and analyzed on BD FACSCanto II (BD Biosciences, CA, USA) using FlowJo software (TriStar, CA, USA). Standardized fluorescence intensities were calculated by dividing the median fluorescence intensities of specific antibodies by the median fluorescence intensities of isotype-matched control. All data were collected from three independent experiments. Statistical significance was determined by paired-samples *t* test.

### Tissue Microarray (TMAs) and Immunohistochemistry (IHC)

TMAs were constructed using automated tissue microarray instrument (ALPHELYS, Plaisir, France). After identifying the H&E-stained slides for optimal tumour tissue, two cylindrical core biopsies (2-mm diameter) were punched from each formalin-fixed, paraffin-embedded tissue blocks and arrayed in recipient TMA blocks (2×3 cm) as previously described [27], [28]. Each TMA block includes 108 tissue cores from 54 patients. Subsequently recipient blocks were cut into 5-µm sections and adhered to the silanized glass slides for IHC staining.

IHC was performed using the Elivision™ super HRP (Mouse/Rabbit) IHC Kit (Maixin-Bio, Fuzhou, China) according to the manufacturer’s instructions. After deparaffinised in xylene and rehydrated in a series of graded alcohols (100%, 95%, 75%), the slides were retrieved antigen in sodium citrate buffer (pH 6.0) in microware. Then the slides were incubated with 3% H_2_O_2_ to block endogenous peroxidase and goat serum to block nonspecific binding. Afterwards, slides were incubated with PD-L1 antibody (diluted 1∶500, Abcam, HK, China) or PTEN antibody (diluted 1∶600, Abcam, HK, China) overnight at 4°C. The next day the slides were incubated with amplification agent (reagent A, Elivision super Kit supply) and polymerase (reagent B, Elivision super Kit supply). Lastly, the slides were stained with DAB and counterstained with hematoxylin (40 seconds). A negative control using antibody dilution as substitute for primary antibodies was performed for each experiment.

### Staining Evaluation

For each spot, a digital image at 2592×1944 pixels resolution at 100× magnification were captured by the Leica DMI 4000B inverted microscope (Leica Micro-systems, Wetzlar, Germany). Immunohistochemical staining of the image was analysed by using the Image Pro-Plus (version 5.0, Media Cybernetics, Silver Spring, USA) introduced by Xavier [29]. In brief, the tumour area was selected as the area of interest (AOI), and the area sum and integrated optical density (IOD) of the AOI were selected as the measurement parameters. PD-L1 and PTEN expression index equalled the quotient between the IOD and the total area of AOI. Finally, the mean expression index for each duplicate was used for statistical analysis.

### Statistical Analysis

Statistical analyses were done using the SPSS v.17.0 (SPSS Inc, Chicago, USA). X-tile software (version 3.6.1, Yale University School of Medicine, New Haven, USA) was used to determine the optimal single cut-point for the survival curve. All P values were two sided and P<0.05 was considered statistically significant.

Associations of PD-L1/PTEN expression with clinical parameters were evaluated using Wilcoxon rank sum test (for two populations that are related) or Kruskal-Wallis test (for more than two populations that are related) because the data of PD-L1/PTEN expression index isn’t consistent with a normal distribution (Shapiro-Wilk test: P<0.001). Overall survival (OS) and metastasis-free survival (MFS) were estimated using the Kaplan-Meier method. Being diagnosed as metastasis during follow-up in the located CRC (pM0) patients was defined as the terminal event for MFS analysis. The associations of PD-L1/PTEN expression with disease outcome were evaluated using Cox proportional hazards regression models. The optimal single cut-point for PD-L1/PTEN expression that separate patients into a group with PD-L1/PTEN higher expression and a group with PD-L1/PTEN lower expression were constructed by X-tile software as previously described [23], [24]. Similar associations and survival analysis were performed in the subset of 39 patients without any PTEN expression, which represents the group in which the confounding role of PTEN is controlled.

## Results

### Not PTEN Loss but IFN-γ induced PD-L1 mRNA Expression in CRC Cell Lines

**Figure 1:**
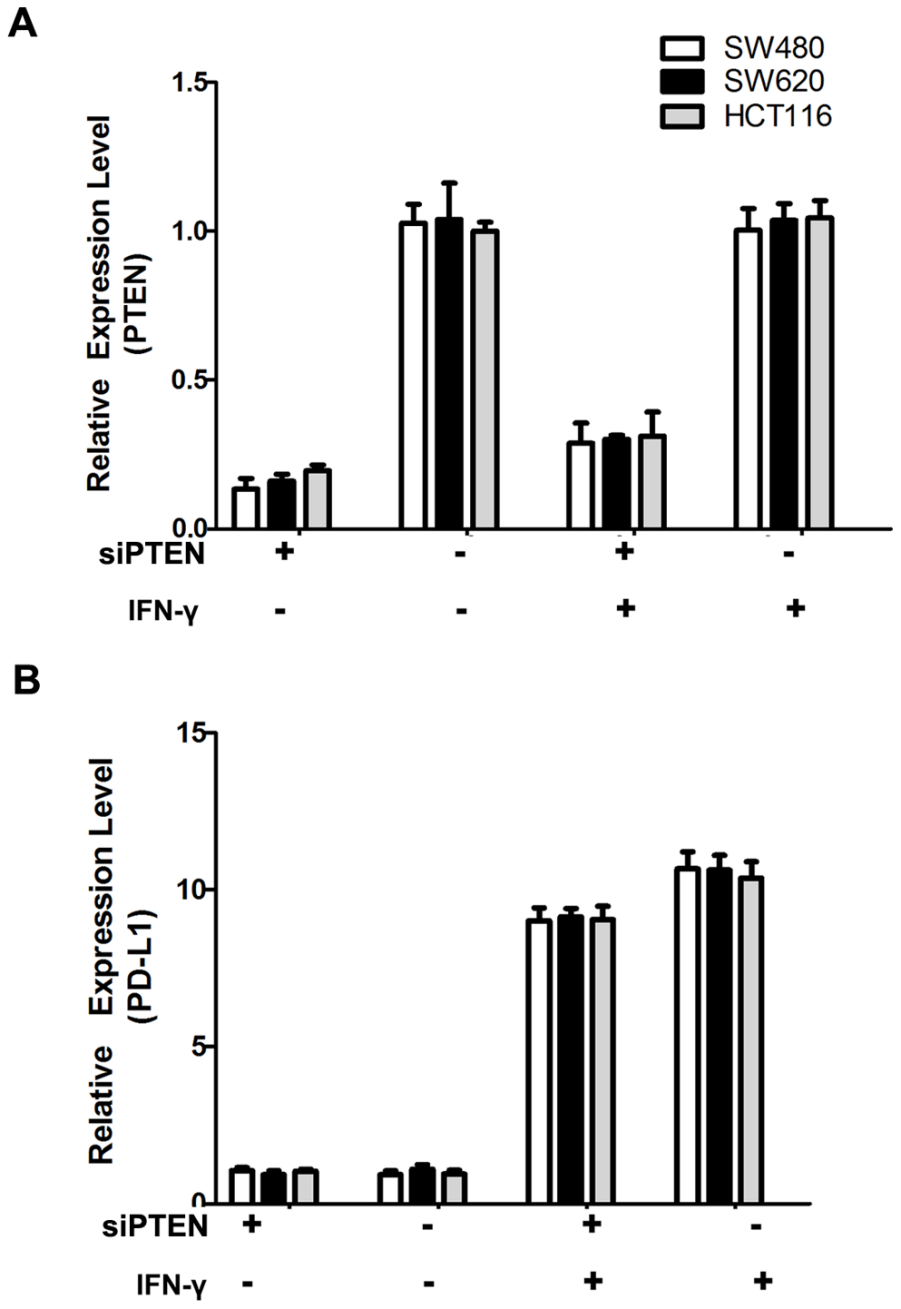
Not PTEN loss but IFN-γ induced PD-L1 mRNA expression in CRC cell lines. (**A**) The relative expression level of PTEN mRNA (detected by qRT-PCR, calculated by 2^−ΔΔCT^ method) in four groups: cells transfected with siRNA PTEN or non-specific sequences in the presence or absence of IFN-γ. (**B**) The relative expression level of PD-L1 mRNA in four groups of cells. Data were collected from three independent experiments. Error bars represent SD.

Both the mRNA level and the protein level of PTEN declined in cells transfected with siRNA PTEN compared with the negative control. The mRNA level of PTEN was reduced down to about 12.4% in SW480, 14.6% in SW620, 22.6% in HCT116 at 48 h post transfection in cells transfected with siRNA PTEN compared with the negative control (Fig. 1. A). There was no significant difference in the PD-L1 mRNA level between the cells transfected with siRNA PTEN and the negative control. By contrast, PD-L1 mRNA level increased significantly in cells which were exposed to IFN-γ compared with the matched-control cells without exposure to IFN-γ (about 8-folds) (Fig. 1. B).

### Both IFN-γ and PTEN Loss Induced PD-L1 Protein Expression in CRC

**Figure 2:**
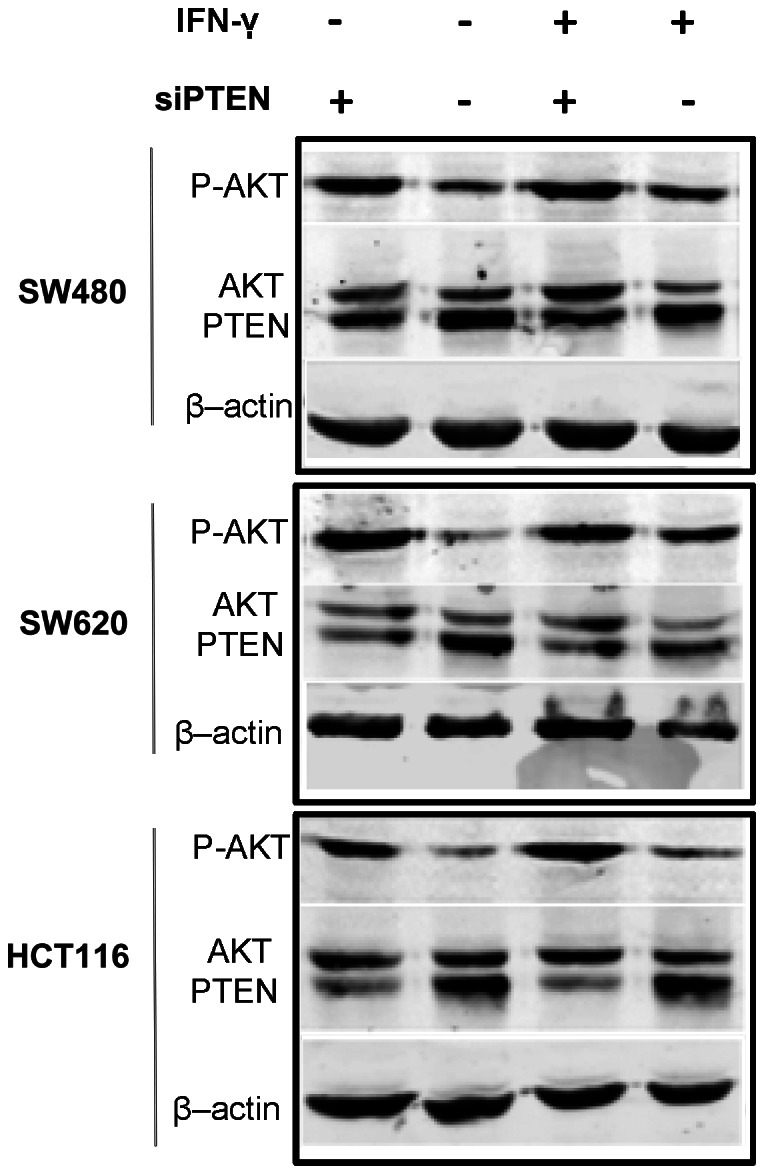
PTEN knockdown increased Akt activation both in the presence and absence of IFN-γ. The protein expression level of PTEN, Akt and phospho-Akt^Ser473^ were determined by Western blotting. β-actin was used to verify equal loading. Representative images are selected from performed experiments repeated in triplicates.

**Figure 3:**
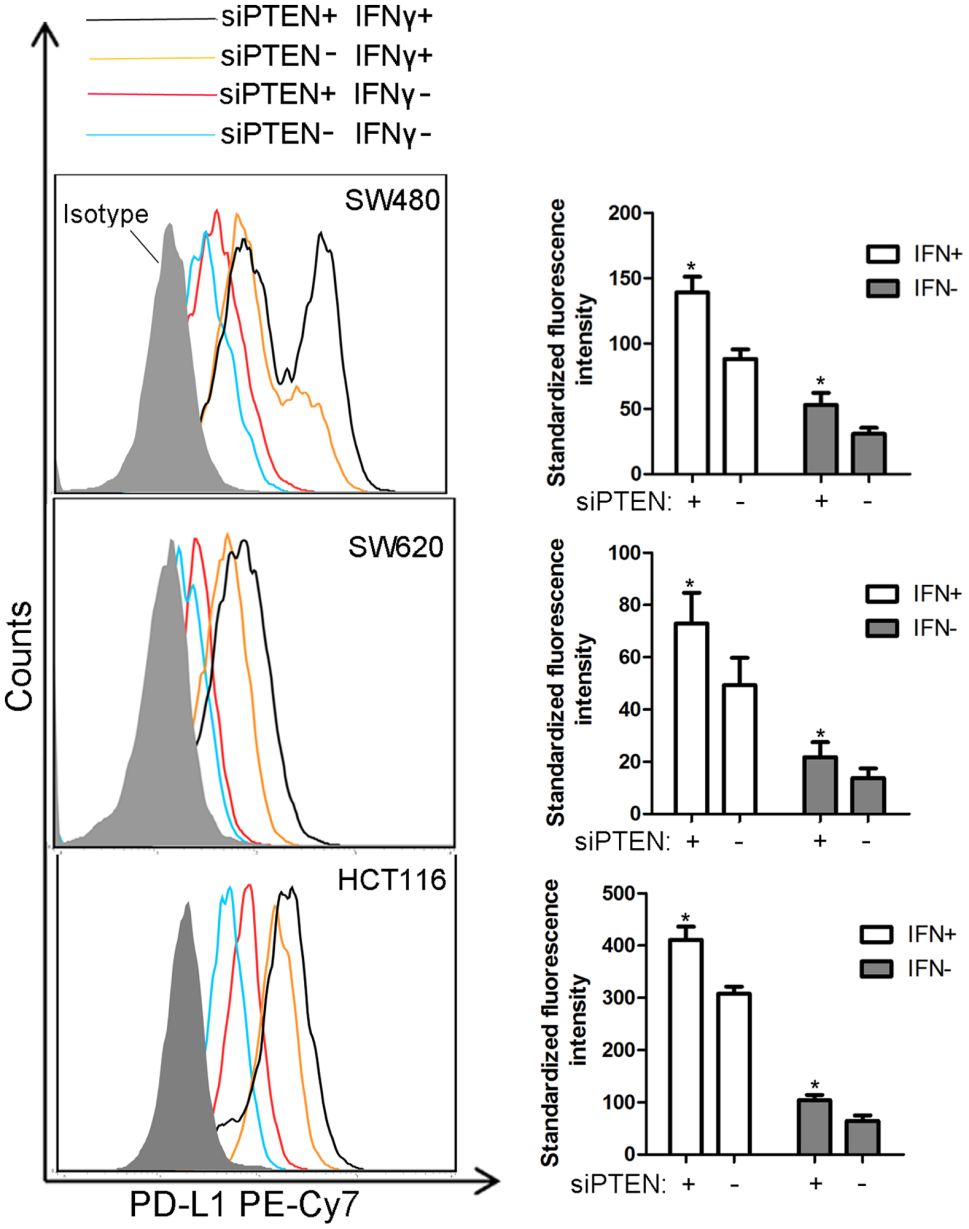
Effect of IFN-γ and PTEN loss on the expression of PD-L1 protein. PD-L1 protein expression level on the surface of SW480, SW620 and HCT116 was determined by flow cytometer: cells transfected with siRNA PTEN (red lines) and cells transfected with non-specific sequences (blue lines) in the absence of IFN-γ; cells transfected with siRNA PTEN (black lines) and cells transfected with non-specific sequences (orange lines) in the presence of IFN-γ; cells stained with isotype-matched control antibody (shaded area). Standard fluorescence intensity of PD-L1 protein was described by histogram in right panel: cells treated (white column) or untreated (gray column) with IFN-γ. Data were collected from three independent experiments. Error bars represent SD. (*P<0.05 by paired-samples *t* test).

We evaluated consequences of PTEN knockdown on Akt activation in the presence/absence of IFN-γ. Phospho-Akt^Ser473^ increased in cells transfected with siRNA PTEN compared with the negative control, regardless of the presence of IFN-γ (Fig. 2). PD-L1 protein expression was significantly increased in cells transfected with siRNA PTEN compared with the negative control (P<0.05). In addition, the presence of IFN-γ did not change this baseline situation (Fig. 3).

### IFN-γ Induced PD-L1 Protein with Two Different Structures in the SW480 Cell Line

**Figure 4:**
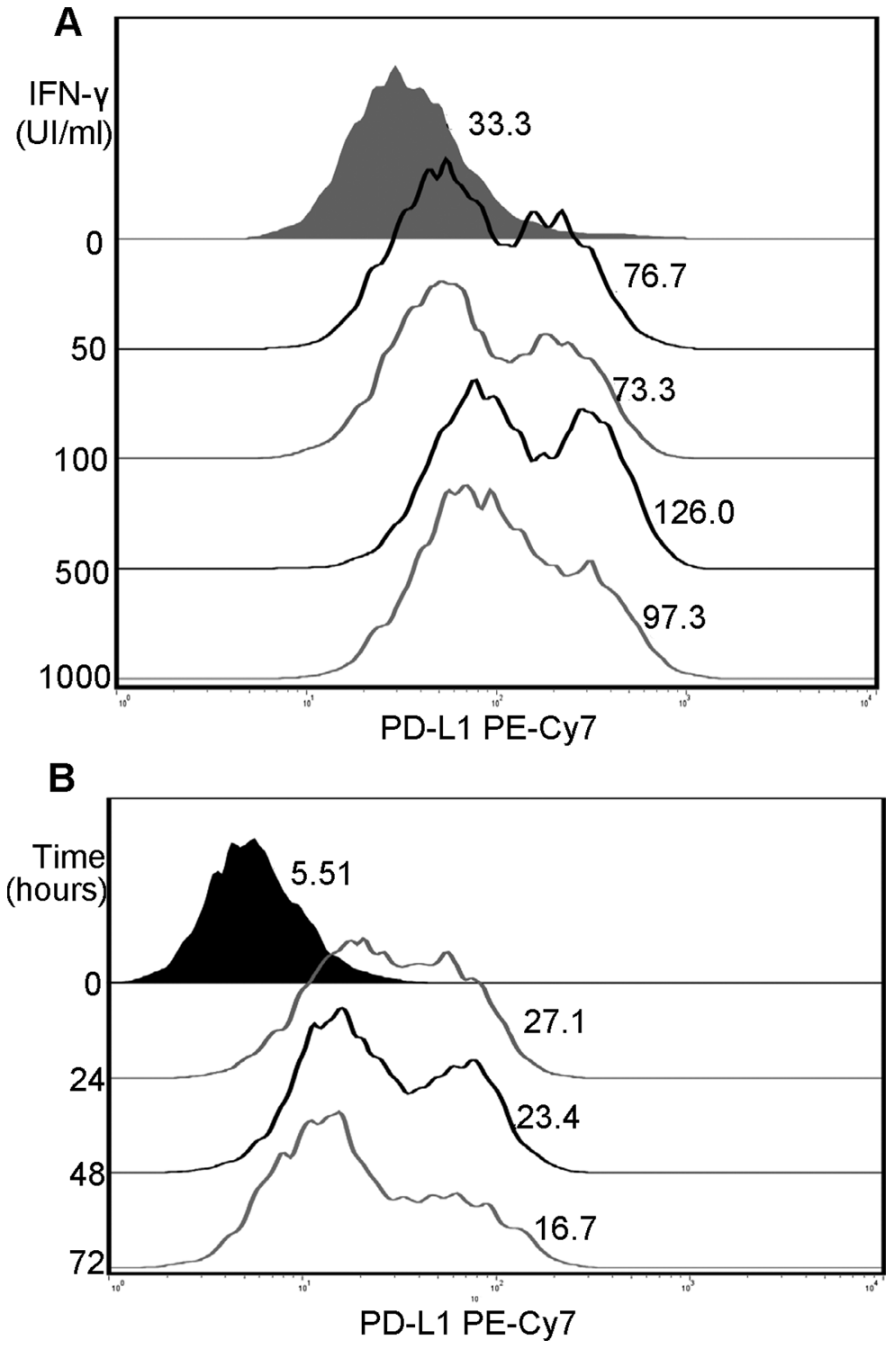
Dosage-course and time-course effect of IFN-γ on PD-L1 in SW480. (**A**) SW480 treated with IFN-γ at indicated concentration (0 IU/ml, 50 IU/ml, 100 IU/ml, 500 IU/ml, 1000 IU/ml). (**B**) SW480 treated with 500 IU/ml IFN-γ for indicted times (0 h, 24 h, 48 h, and 72 h). Shaded area represents SW480 without treatment of IFN-γ, and numbers next to peaks represent standard fluorescence intensity of PD-L1. Representative images are selected from performed experiments repeated in triplicates.

The flow cytometry histograms showed two peaks in SW480 cells treated with IFN-γ (500 IU, 72 hours), while there was only one single distinct peak in untreated group. In addition, only one single distinct peak was observed in SW620 cells and HCT116 cells both in the presence and absence of IFN-γ (Fig. 3). To determine whether these two peaks were time-dependent or dosage-dependent, we treated SW480 cells with different dosage of IFN-γ for 48 hours. PD-L1 protein was maximally increased at 500 IU/ml IFN-γ. Time-course effect of IFN-γ on the PD-L1 protein was determined thereafter. SW480 was treated with 500 IU/ml IFN-γ for 0, 24, 48 and 72 hours. The results showed that in the presence of IFN-γ, regardless of the dosage of IFN-γ and the time of treatment, PD-L1 protein with two different structures was recognized on the surface of SW480 cells (Fig. 4).

### Patient Follow-up

Among the 404 patients, 5 patients (<3%) had been lost at the end of follow-up, 110 patients (27.2%) had died at a median follow-up time of 2.7 years (range: 0.1–9.1 years), and the remaining 288 patients had a median follow-up time of 5.8 years (range: 0.1–10.2 years). When IHC was performed, the tissue of 57 patients was washed off the glass slides and the remaining 347 patients had immunohistochemical data. There was no difference in overall survival between patients with and without immunohistochemical data (P = 0.716, log-rank test). There was also no significant difference in metastatic progression (diagnosed as metastasis after surgery) rates between patients with and without immunohistochemical data (12.4% and 14.0%, P = 0.682).

### Correlation of PD-L1/PTEN Expression and Clinical Parameters

**Figure 5:**
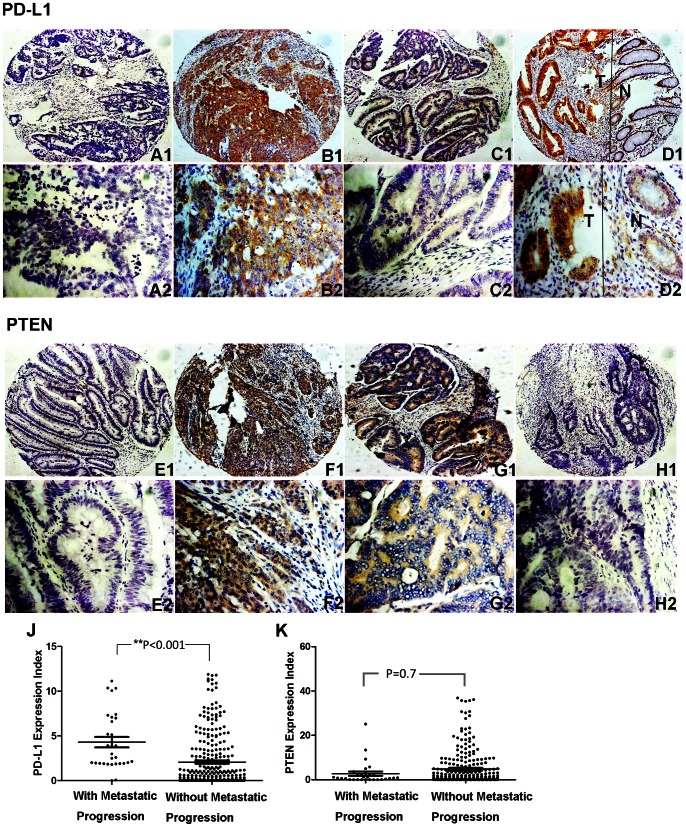
Representative Immunohistochemical results of PD-L1/PTEN for patients with and without metastatic progression. (**A_1_, A_2_, E_1_, E_2_**) Negative control; (**B_1_, B_2_, F_1_, F_2_**) Specimens from the same patient with metastatic progression; (**C_1_, C_2_, G_1_, G_2_**) Specimens from another patient without metastatic progression; (**D_1_, D_2_**) A typical representative which showed that PD-L1 expression is elevated in the tumor area (T) compared with those of adjacent normal mucosa (N); (**H_1_, H_2_**) Specimens from patient without any PTEN expression; (**J, K**) Statistical analysis demonstrated that PD-L1 expression is increased in the patients with metastatic progression compared with those without metastatic progression, while such statistical analysis of PTEN wasn’t significant. (Two up panels, stained with PD-L1 antibody; two down panels, stained with PTEN antibody), (A_1_–H_1_, ×100; A_2_–H_2_, ×400).

The immunohistochemical staining of PD-L1 located in cell membrane and endomembrane system had an expression index ranged from 0 to 15.9 (median: 2.33) (Fig. 5. A_1_–D_1_, A_2_–D_2_). PTEN was located in cytoplasm with an expression index ranged from 0 to 54.7 (median: 5.57) (Fig. 5. E_1_–G_1_, E_2_–G_2_).There was 39 patients that had no PTEN expression (Fig. 5. H_1_, H_2_).

**Figure 6:**
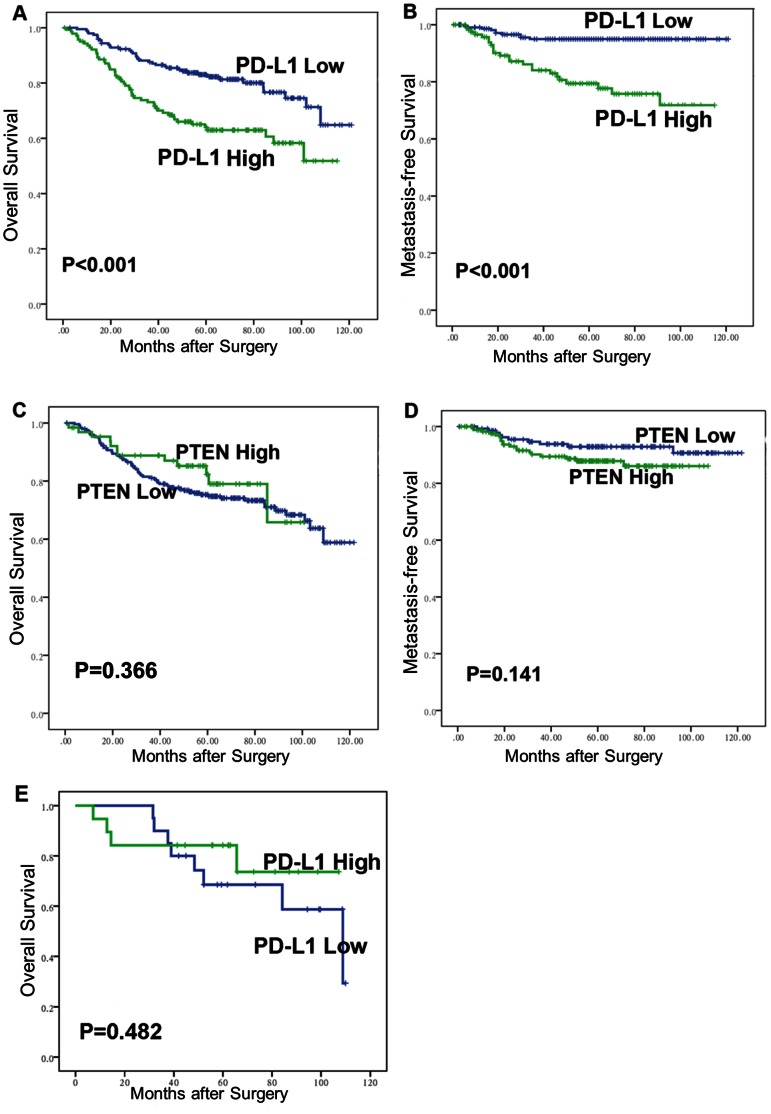
Association of PD-L1/PTEN expression with overall survival (OS) and metastasis-free survival (MFS) in 347 CRC patients. (**A, B**) The OS and MFS were significantly lower in PD-L1-higher-expression patients compared with PD-L1-lower-expression patients (both P<0.001). (**C, D**) The OS and MFS were not significantly different between the PTEN-higher-expression patients and the PTEN-lower-expression patients (OS, P = 0.366; MFS, P = 0.141). (**E**) In the subset of 39 patients with complete PTEN loss, PD-L1 expression was no longer associated with the OS (P = 0.482).

As shown in Table 2, patients with higher PD-L1 expression were more likely to exhibit distant metastases (pM) (P<0.01), adverse pathologic features (2007 TNM classification) (P<0.01), and metastatic progression (P<0.001) (Fig. 5. J), and PD-L1 expression was correlated with PTEN expression to some extent (P<0.001). There was no statistically significant difference between genders, age, tumor location, primary tumor classification (pT), regional lymph node involvement (pN), or recurrence after surgery. Conversely, PTEN expression had no significant association with the primary variables (pT: P = 0.221; pN: P = 0.131; pM: P = 0.525; TNM stage: P = 0.337; recurrence: P = 0.397; metastatic progression: P = 0.710). The overall survival and metastasis-free survival was also not associated with PTEN expression (P = 0.366, P = 0.141 respectively) (Fig. 6. C, D).

**Table 2:** Correlation between PD-L1 expression and clinical parameters in 347 colorectal cancer patients.

Variable	n	PD-L1 expression	P value
**Genders**			0.722
Female	187	0.647(0.184, 3.916)	
Male	160	0.993(0.116, 2.869)	
**Age**			0.963
<65	209	0.820(0.016, 3.670)	
≥65	138	0.881(0.018, 3.086)	
**Tumor location**			0.293
Colon	170	0.552(0.012, 3.481)	
Rectum	177	0.920(0.043, 3.542)	
**Primary tumor (pT)**			0.566
T1+ T2	64	1.001(0.024, 3.941)	
T3+ T4	283	0.784(0.016, 3.454)	
**Regional lymph node (pN)**			0.331
N0	199	0.594(0.013, 3.524)	
N1+N2	148	1.101(0.027, 3.670)	
**Distant metastasis (pM)**			<**0.01**
M0	319	0.594(0.139, 3.037)	
M1	28	2.851(1.628, 6.209)	
**TNM stage**			
I	57	0.569(0.009, 2.285)	<**0.01**
II	138	0.578(0.016, 3.550)	
III	129	0.784(0.015, 2.975)	
IV	33	2.851(1.628, 6.210)	
**Recurrence after surgery**			
Yes	20	1.454(0.115, 2.711)	0.725
No	327	0.820(0.016, 3.524)	
**Metastatic progression**			<**0.001**
Yes	34	3.419(1.930, 7.165)	
No	306	0.536(0.010, 2.936)	
**PTEN expression**			<**0.001**
<0.867 (median)	157	1.869(0.298, 5.207)	
≥0.867	157	0.115(0.000, 2.080)	

Note: Data of PD-L1 expression are described with median (P25, P75). Wilcoxon rank sum test (Kruskal-Wallis test) for all analysis.

### Survival Analysis

Analysis of the X-tile software reveals that 1.49 was the optimal cut-point that separated patients into a group with PD-L1 higher expression and a group with PD-L1 lower expression. The Kaplan-Meier curve and log-rank test showed that the overall survival rate of patients with lower PD-L1 expression was significantly higher than the patients with higher PD-L1 expression (Fig. 6A, χ2 = 13.964, P<0.001). The 5-year OS rate was 62.9% for patients with higher PD-L1 expression and 81.1% for patients with lower PD-L1 expression. In multivariate analysis by Cox proportional hazards model, PD-L1 was not an independent prognostic factor for CRC after adjusting for the variables that meet PH assumption (age, tumor location, differentiation grade, pT, pN, pM, metastatic progression) (P = 0.548). However, if variables that significantly associated with PD-L1 expression (pM, metastatic progression) were not adjusted for in multivariate analysis, PD-L1 expression became a significant predictor for CRC (P<0.01) (Table 3).

**Table 3:** Cox multivariate analysis of factors associated with overall survival with and without adjusting for the variables that significantly associated with PD-L1 expression.

Variable	Adjusted for PD-L1-associated variables			Unadjusted for PD-L1-associated variables		
	RR	95%CI	P Value	RR	95%CI	P Value
**Age**	1.776	1.122–2.811	<0.05	1.363	0884–2.102	0.16
**Tumor location**	0.552	0.346–0.882	<0.05	0.503	0.381–0.796	<0.01
**Differentiation grade**	0.561	0.314–1.001	<0.05	0.374	0.213–0.656	<0.01
**Primary tumor (pT)**	1.76	0.839–3.691	0.135	2.351	1.176–4.702	<0.05
**Regional lymph node (pN)**	1.898	1.186–3.037	<0.01	2.053	1.303–3.236	<0.05
**Distant metastasis (pM)**	7.808	4.285–14.229	<0.001			
**Metastatic progression**	2.578	1.390–4.782	<0.01			
**PD-L1 expression index**	1.174	0.696–1.979	**0.548**	2.07	1.342–3.193	<**0.01**

As shown in our results, PD-L1 inclined to associate with the metastasis variables. So to further determine the association of PD-L1 with cancer metastatic progression, we studied the subset of 310 patients with localized CRC (pM0), of whom 28 (9.03%) progressed to distant metastases at a median follow-up of 5.14 years (range, 0.06–10.14 years). In this subset, PD-L1 expression was significantly associated with metastasis-free survival (MFS) (Fig. 6B, χ2 = 21.444, P<0.001). The 5-year MFS rate was 99.5% for PD-L1-lower-expression patients and 72.1% for PD-L1-higher-expression patients. In addition, Cox analysis revealed that PD-L1 expression was a significant predictor for metastatic progression (RR: 1.165; 95% CI: 1.072–1.268; P<0.001).

Interestingly, in the subset of 39 PTEN-no-expression patients, in which the confounding effect of PTEN on PD-L1 is controlled, PD-L1 expression was no longer associated with distant metastasis (P = 0.102), TNM stage (P = 0.634), and survival analysis showed that there was no significant difference in the overall survival between the PD-L1-higher-expression group and PD-L1-lower-expression group (Fig. 6E, χ2 = 0.494, P = 0.482).

## Discussion

PD-L1 has been one of the major subjects of tumour biomarker research in the past decade. A large number of studies have demonstrated a correlation between tumor aggressiveness and PD-L1 protein expression. In the present study, we also provided strong evidence that a higher level of PD-L1 expression in CRC facilitates advancement of tumor stage and metastatic progression. The overall survival rate of patients with lower expression of PD-L1 was significantly higher than that of patients with higher expression of PD-L1. In addition, PD-L1 expression was also significantly associated with metastasis-free survival in the 310 located CRC patients. However, PD-L1 expression was not significantly associated with unfavorable prognosis after adjusting for metastasis and metastatic progression by multivariate survival analysis. This is inconsistent with the previous observation that PD-L1 expression remains associated with unfavorable prognosis by multivariate analysis in renal cell carcinoma patients [6]. One explanation for this discrepancy may be the different variables which are adjusted for in the multivariate model. In the previous study, the metastatic progression variable (diagnosed as metastasis after surgery) was not adjusted for in the multivariate survival analysis. It’s well known that metastasis is the primary cause of death in CRC patients; so once we adjusted for this death-related variable, PD-L1 was no longer an independent prognostic factor for CRC.

Evidences from the literatures and our research showed that PD-L1 overexpression is closely associated with cancer progression (especially with metastasis progression), making PD-L1 a promising biomarker and therapeutic targets for tumors. However, the mechanism by which PD-L1 enhances cancer progression is poorly understood. As described in literature, these associations may be attributed to the well-recognized “molecular shield” provided by PD-L1. It was reported that PD-L1 provides a “shielding” effect to protect tumor cells from lysis by cytotoxic T cells. In addition, PD-L1 was found to have an anti-apoptotic effect on tumor cells [3], [30]. However, an in *vivo* study showed that transgenic expression of PD-L1 in a PD-L1-deficient naive mouse did not change its tumourigenicity [31]. In the multicentre phase I trial, Brahmer et al declared that antibody-mediated blockade of PD-L1 induces durable tumor regression and prolongs stabilization of disease in patients with select advanced cancers. Unfortunately, colorectal cancer is not one of these select cancers [32]. We wondered whether the ability of destroying the “molecular shield” can provide adequate basis for this clinical association in CRC. Andrew T Parsa also suggested that the extent to which PD-L1 protein expression directly affects cancer progression remains to be determined [14].

In this study, to identify additional indirect effects of PD-L1 expression on cancer progression, we studied the signaling pathways involved in the regulation of PD-L1 expression in CRC. Previous studies suggested that the signaling pathways varied among different tumors. It has been found that PTEN/PI3K/Akt pathway may be important for the induction of PD-L1 expression in gliomas. However, research in multiple myeloma (MM) did not support the role of PTEN/PI3K/Akt pathway in inducing PD-L1 expression. Instead, it indicated that IFN-γ-inducible PD-L1 expression is induced through the MyD88/TRAF6 and MEK/ERK pathways. In the present study, our data supported a role for PTEN in inducing PD-L1 expression. PD-L1 protein but not mRNA level was elevated in CRC cells transfected with siRNA PTEN compared with the negative control. Moreover, IFN-γ, the main inducer of PD-L1 expression, did not affect the capacity of PTEN to regulate PD-L1 expression. It is likely that PTEN and IFN-γ regulate the PD-L1 expression through independent signaling pathways. PTEN loss changes the level of PD-L1 protein through the regulation of translation, while IFN-γ modulates PD-L1 levels through the regulation of both transcription and translation. Moreover, IFN-γ modifies the protein structure of PD-L1 in certain types of CRC cell lines (e.g. SW480).

It is well known that PTEN participates in various central processes of cancer development, and that it is an important gene for cancer diagnosis and prognosis. In support of previous research, our study shows that PD-L1 expression correlates to some extent with PTEN expression in CRC (P<0.001) [14]. It is possible that cancer progression and PD-L1 protein expression are independently related to the PTEN loss in cancer cells, and the clinical effect of PD-L1 may be, at least in part, attributed to an association between PTEN loss and PD-L1 expression. To verify this hypothesis, we analyzed whether PD-L1 expression still correlates with poor prognosis and metastatic progression after eliminating the confounding factor. In order to analyse the clinical effect of PD-L1 without concern of whether this clinical effect was attributed to different PTEN levels, thirty-nine patients with PTEN complete loss were selected in which PTEN expression was exactly the same. The results showed that once PTEN expression is controlled, PD-L1 expression is no longer correlated with distant metastasis, TNM stage, and overall survival, supporting the hypothesis that the association of PD-L1 expression with poor survival and metastatic progression may be partly conferred by PTEN loss. However, the sample size of this analysis is rather small (only 39 patients). To obtain more powerful evidence, studies with lager sample size are in urgent need.

The regulation mechanism of PD-L1 may differ among different types of tumors. It may result in the phenomenon that PD-L1 inhibition alone does not appear to help all patients with cancer, which was showed in the multicentre phase I trial of anti-PD-L1 [32]. Identification of all the counter-receptors in these pathways and development of combinatorial therapeutic strategies could lead to reliable and consistent clinical efficacy. For CRC patients with PTEN mutation, combinatorial therapeutic strategies that target PD-L1, PTEN and other well-defined molecules may be a better option in preclinical and clinical settings. In addition, selection of patients through the use of predictive biomarkers may be reasonable strategies, and predictive biomarkers should be selected depend on the form of CRC, more specifically; BRAF appears to be a strong prognostic factor for OS after relapse, particularly in MS-L/S stage II patients [33]. Given the observation that CRC patients with overexpression of PD-L1 are at significantly higher risk of metastatic progression, PD-L1 appears to be a promising prognostic factors for mCRC.

In the present study, what was most surprising was the clinical effect of PTEN. Although accumulating evidence has strongly suggested that PTEN is a crucial factor in various central processes of cancer development, the association between PTEN expression and clinical parameters in CRC is still controversial. A study with 482 colorectal adenocarcinomas revealed that PTEN is associated with poor overall survival and disease-free survival (P = 0.030 and P = 0.046, respectively) [21], while two other studies observing 76 and 125 CRC patients found no correlation between PTEN status and patient survival [34], [35]. In addition, in all three studies, no significant relation between PTEN expression and tumor grade, TNM stage, lymphatic invasion, and liver metastasis could be found. In our study, we also could not find any association between PTEN expression and clinical parameters. This was intriguing considering that PTEN is such a crucial factor in various central processes of cancer development. Some researchers demonstrated that PTEN is localized to both the cytoplasm and the nucleus and shuttles between each other by a variety of mechanisms. The mechanism involved in this shuttle depends on the cell types and cellular environment [36]. So the clinical manifestations of PTEN function are, at least in part, determined by its subcellular localization. It is not applicable to determine the clinical manifestations of PTEN using nucleus or cytoplasm immunohistochemical staining separately. That is the reason why we only select patients with PTEN complete loss to explore whether PD-L1 still correlates with clinical parameters when the clinical manifestations of PTEN are controlled in the same level.

Taken together, we determined that PTEN loss and IFN-γ regulate the PD-L1 expression independently in CRC. Our study additionally suggested that CRC patients with overexpression of PD-L1 are at significantly higher risk of metastatic progression and mortality. This clinical manifestation may be not only affected directly by the recognized “molecular shield” provided by PD-L1, but also affected indirectly by the correlation between PD-L1 protein expression and PTEN loss. Our findings will contribute to a more comprehensive understanding of the prognosis and therapeutic value of PD-L1 in tumours.
